# Neoadjuvant Immunotherapy in Cutaneous Squamous Cell Carcinoma: Systematic Literature Review and State of the Art

**DOI:** 10.3390/cancers17040637

**Published:** 2025-02-14

**Authors:** Marco Spadafora, Alessia Paganelli, Margherita Raucci, Shaniko Kaleci, Ketty Peris, Stefania Guida, Giovanni Pellacani, Caterina Longo

**Affiliations:** 1 Azienda Unità Sanitaria Locale, IRCCS di Reggio Emilia, Skin Cancer Center, 42122 Reggio Emilia, Italy; spadafora.marco91@gmail.com (M.S.); margherita.raucci@ausl.re.it (M.R.); 2 Department of Surgery, Medicine, Dental Medicine and Morphological Sciences, University of Modena and Reggio Emilia, 41124 Modena, Italy; shaniko.kaleci@unimore.it; 3 IDI-IRCCS Istituto Dermopatico dell’Immacolata, 00167 Rome, Italy; alessia.paganelli@gmail.com; 4 Dermatology Unit, Department of Surgical and Medical Sciences, Fondazione Policlinico Universitario Agostino Gemelli IRCCS, 00168 Rome, Italy; ketty.peris@unicatt.it; 5 School of Medicine, Vita-Salute San Raffaele University, 20132 Milan, Italy; guida.stefania@hsr.it; 6 Dermatology Clinic, IRCSS San Raffaele Hospital, 20132 Milan, Italy; 7 Department of Clinical Internal, Anesthesiological and Cardiovascular Sciences, Dermatology Clinic, Sapienza University of Rome, 00185 Rome, Italy; pellacani.giovanni@uniroma1.it; 8 Department of Dermatology, University of Modena and Reggio Emilia, 42122 Modena, Italy

**Keywords:** neoadjuvant, immunotherapy, skin cancer, squamous cell carcinoma, therapy, non-melanoma skin cancer, cSCC, oncology

## Abstract

Background/Objectives: Cutaneous squamous cell carcinoma (cSCC) is a prevalent skin cancer with increasing incidence worldwide. High-risk cSCCs often require extensive surgical treatments, which can impair anatomical function and aesthetics. Neoadjuvant immunotherapy, particularly immune checkpoint inhibitors (ICIs) such as cemiplimab and pembrolizumab, has emerged as a promising approach to enhance tumor control and surgical outcomes. We performed a systematic review to evaluate the efficacy and safety of neoadjuvant immunotherapy in high-risk cSCC. Methods: A systematic review and proportional meta-analysis focusing on neoadjuvant immunotherapy for cSCC were conducted following PRISMA guidelines. MEDLINE and Scopus databases were searched up to September 2024 using predefined terms. Recorded findings were pathological/radiological response, 1-year disease-free survival (DFS), and overall survival (OS). Data extraction and risk-of-bias assessment were independently performed by two reviewers. Statistical analysis included fixed- and random-effects models, with heterogeneity assessed using Cochran’s Q statistic and the I² index. Results: Nine studies met the inclusion criteria. The pooled pathologic response rate was 72.2% (95% CI: 57.7–84.6), and the radiological response rate was 54.8% (95% CI: 38.6–70.5), with moderate heterogeneity. Pooled 1-year DFS and OS proportions were 91.1% (95% CI: 85.0–95.3) and 90.6% (95% CI: 85.1–95.0), respectively, demonstrating homogeneity across studies. Adverse events were consistent with previously reported immune-related toxicities, with rare severe events. Conclusions: Neoadjuvant immunotherapy is a promising therapeutic strategy for high-risk cSCC, with high pathologic response rates and high survival outcomes. However, standardization of treatment protocols and further trials are needed to optimize efficacy, ensure safety, and assess long-term benefits.

## Simple Summary

Skin cancer, particularly cutaneous squamous cell carcinoma (cSCC), is becoming more common, and advanced cases often require complex surgery that can affect both aesthetics and anatomical functionality. Neoadjuvant immunotherapy, which enhances the immune system to fight cancer before surgery, has recently shown promising results in improving outcomes. This systematic review evaluated studies on this topic to describe the efficacy of neoadjuvant immunotherapy in high-risk cSCC. Our review suggests that this approach not only reduces tumor burden but also leads to higher survival rates. However, the development of standardized treatment methods and further studies on this topic are needed to achieve better results in patient therapeutic management and a higher quality of life.

## 1. Introduction

Cutaneous squamous cell carcinoma (cSCC) is the second most common type of skin cancer globally, accounting for 20% to 50% of all skin cancers [1]. It was estimated 2.4 million new cases and approximately 56,000 deaths are reported every year globally [2]. Over the past three decades, the incidence of cSCC has increased by more than 200%, largely attributed to population aging, cumulative sun exposure, and immunosuppression [2]. A subset of cSCCs is characterized by locoregionally advanced disease or pathological features such as deep invasion, perineural invasion, and recurrent tumors, which increase the risk of locoregional spread and distant metastases. In such high-risk cases, adjuvant radiation therapy (RT) is commonly used to improve disease control [3]. Despite the efficacy of surgery and RT, these approaches can result in substantial functional and cosmetic impairments, particularly in cSCC involving the head and neck area. In this setting, the neoadjuvant approach finds its best application with regard to cSCCs. Neoadjuvant immunotherapy, as demonstrated in non-small cell lung cancer, melanoma, and colorectal cancer, has shown potential to enhance disease control, reduce tumor burden, and improve surgical outcomes, offering a significant advantage over conventional treatments [4,5,6,7].

For patients with metastatic or locally advanced cSCC who are ineligible for curative surgery or RT, immune checkpoint inhibitors (ICIs) targeting programmed cell death protein 1 (PD-1), such as cemiplimab and pembrolizumab, have recently emerged as effective therapeutic options [8,9,10]. Significant response rates of ICIs are likely due to the high tumor mutational burden, which was previously characterized in cSCC mutational studies [11,12]. The promising outcomes of ICIs in advanced cSCC created greater interest in evaluating their use as neoadjuvant therapies in resectable disease. Avoiding more aggressive treatments, the use of neoadjuvant immunotherapy might lead to an improved quality of life for patients with advanced skin cancers who are in a fragile state [13].

The aim of the present study is to systematically review the available literature on neoadjuvant immunotherapy on cSCC.

## 2. Materials and Methods

**Figure 1:**
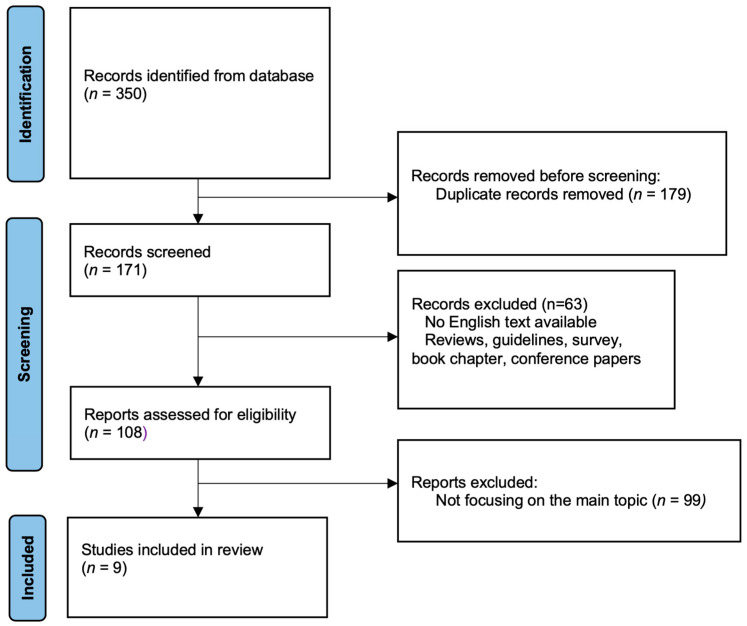
Workflow diagram describing the systematic selection of studies for inclusion in the present review (PRISMA flow-chart).

This systematic review was based on the PRISMA guidelines [14]. A flow chart diagram is presented in Figure 1, and the PRISMA Checklist is presented as Supplementary Materials.

### 2.1. Search Strategy

A search was conducted in the MEDLINE and Scopus electronic databases from inception to the present (up to September 2024). The detailed search strategy used the following terms: ‘cutaneous squamous cell carcinoma’ [Title/Abstract]) AND ‘neoadjuvant [Title/Abstract]’ OR ‘cSCC’ [Title/Abstract] AND ‘neoadjuvant [Title/Abstract]’. The terms were adapted for databases as appropriate. Only journal articles were taken into consideration, while books and book chapters were excluded. Articles without full text electronically available and/or English translation were also excluded. Review articles, guidelines, surveys, book chapters, and conference papers were not included. Only journal articles specifically focusing on the use of neoadjuvant immunotherapy in cSCC were taken into consideration. Studies focusing on neoadjuvant therapies other than immunotherapy, such as chemotherapy, radiation therapy, or targeted therapy, were excluded.

### 2.2. Study Population—Selection

The following PICO (Population, Intervention or exposure, Comparison, Outcome) elements were applied as inclusion criteria for the systematic review: (i) Population: patients diagnosed with high-risk cSCC; (ii) Intervention: use of neoadjuvant immunotherapy; (iii) Comparison: standard of therapy; (iv) Outcome: pathological- and/or radiological response rate and disease-free survival and/or overall survival.

### 2.3. Data Extraction

For studies fulfilling the inclusion criteria, two independent reviewers (M.S. and A.P.) extracted data in a standardized and predefined form. If reviewers identified a title or abstract as potentially significant, it was forwarded for in-depth text analysis, and any disagreements that emerged during this process were resolved through a discussion between the two screeners, with a senior investigator’s assistance when necessary (CL). The following data were collected for each paper: first author and year of publication, study design, sample size, gender, median age, disease feature, drug dosage, pathological response rate (complete or major), radiological response rate (complete or partial), 1-year disease-free survival (DFS), overall survival (OS), adverse events. All co-authors reviewed the final table and figures.

We assessed the risk of bias in individual studies using the Risk-of-bias VISualization (robvis) (version 0.3.0) [15].

### 2.4. Statistical Analysis

We conducted a proportional meta-analysis using MedCalc version 14.8.1. The Freedman-Tukey (square root arc-sine) transformation was applied to calculate the weighted overall proportion [16,17].

Proportions (expressed as percentages) with 95% confidence intervals (CIs) for symptoms identified in each study were included in the meta-analysis. The overall proportion with 95% CI was calculated using both fixed-effects and random-effects models.

The fixed-effects model assumes that all included studies share a common effect, with the summary effect representing the weighted estimate of these similar effects. In contrast, the random-effects model assumes that the effect size varies among studies, providing a weighted average of the reported effects across studies.

A forest plot was used to graphically represent the meta-analysis results. For each included study, the effect size and 95% CI were displayed. The forest plot also depicted the weighted effect size of each symptom with its corresponding 95% CI. The size of the markers (squares) indicates the weight of each study, with smaller studies contributing less weight. The overall effect is represented as a diamond, where the width reflects the precision, and the position indicates the summary estimate.

Heterogeneity among studies was assessed using Cochran’s Q statistic and the I² index. Heterogeneity was considered statistically significant when *p* < 0.01 for the Q statistic, indicating that the observed variance exceeded the expected variance.

The I² index was interpreted as follows: I^2^ = 0–25%, Homogeneous; I^2^ = 25–50%, Moderate heterogeneity; I^2^ = 50–75%, Large heterogeneity; I^2^ = 75–100%, Extreme heterogeneity.

The I^2^ index was calculated as I^2^ = 100% × (Q − df)/QI2 = 100% × (Q − df)/Q, where df represents the degrees of freedom

## 3. Results

The bibliographic research allowed us to identify 350 publications. 179 papers were not included after duplicate removal, while 63 papers were excluded because of the study design (reviews, guidelines, surveys, book chapters, conference papers). Excluding papers not focusing on the main topic, 9 papers were considered for this review (Figure 1) [11,18,19,20,21,22,23,24,25]. Two of the reported publications were follow-up papers of previous studies that were included in the analysis. [11,20] An *erratum* of a paper by Ferrarotto et al. [21] was also reviewed but not included [21]. Table 1 provides a more comprehensive overview of the included articles. The majority of the data focused on cemiplimab; however, treatment schedules were not standardized, either in terms of duration or time to surgery. Cemiplimab was administered at 350 mg every 3 weeks, with durations ranging from two doses to up to a year of therapy before scheduled surgery. Pembrolizumab was given at 200 mg every 3 weeks or 400 mg every 6 weeks for up to a year of therapy. Further, studies reported different time-frames between neoadjuvant therapy and surgery, between days 75 and 10 [24] or a median time of 30 days (range, 21–50) [21].

Adverse events were not homogeneously reported in all studies. A single study described a small percentage (18%) of ≥grade 3 events, with 4 lethal events [24]. Still, only one was considered by the investigator as possibly related to treatment because of the exacerbation of congestive heart failure.

**Figure 2:**
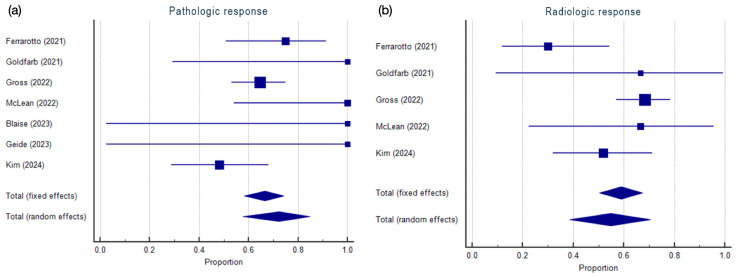
Proportional meta-analysis of included studies for pathological and radiological response. Markers represent grouped effects. The position of the diamond represents the estimated effect size, and the width of the diamond reflects the precision of the estimate. (**a**) Aggregate percentage of pathologic response. (**b**) Aggregate percentage of radiologic response [18,19,21,22,23,24,25].

The results of the proportional meta-analysis for pathologic and radiologic responses, including the combined proportion (95% CI), are summarized in Table 2, with estimates of the overall proportion shown in the Forest Plot (Figure 2). The overall proportion for pathologic response was 72.2% (57.7–84.6), demonstrating significant results and large heterogeneity (Q = 12.4, df = 6, I^2^ = 51.7%, *p* = 0.05). For radiological response, the overall proportion was 54.8% (38.6–70.5), indicating significant and large heterogeneity (Q = 10.4, df = 4, I^2^ = 61.8%, *p* = 0.033).

**Figure 3:**
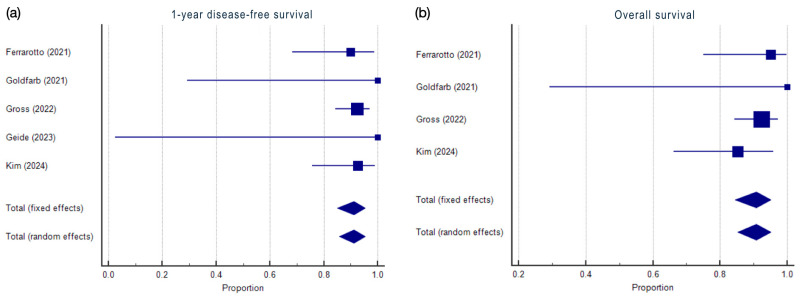
Proportional meta-analysis of included studies for 1-year disease-free survival and overall survival. Markers represent grouped effects. The position of the diamond represents the estimated effect size, and the width of the diamond reflects the precision of the estimate. (**a**) Aggregate percentage of 1-year disease-free survival. (**b**) Aggregate percentage of overall survival [18,21,22,24,25].

The results of the proportional meta-analysis for 1-year DFS and OS, including the combined proportion (95% CI), are summarized in Table 2, with estimates of the overall proportion shown in the Forest Plot (Figure 3). The overall proportion for 1-year DFS was 91.1% (85.0–95.3), demonstrating not significant and homogeneous (Q = 0.34, df = 4, I^2^ = 0.00%, *p* = 0.98). For OS, the proportion was 90.6% (85.1–95.0), indicating not significant and homogeneous (Q = 1.5, df = 3, I^2^ = 0.0%, *p* = 0.68).

## 4. Discussion

This meta-analysis highlights promising outcomes in the context of neoadjuvant immunotherapy for high-risk cSCC. Most of the studies included in the analysis reported a higher percentage of male participants, which aligns with the greater prevalence of cSCC exposure [1].

The findings of this study have important implications for the use of neoadjuvant therapies, particularly ICIs, in the treatment of cSCC. The overall pathologic response rate and radiological response rate emphasize the significant potential of ICIs, such as cemiplimab and pembrolizumab, to reduce tumor burden prior to surgery. These response rates suggest that neoadjuvant immunotherapy may improve surgical outcomes and potentially reduce the need for extensive resections, which is particularly relevant for cSCC cases involving cosmetically and functionally sensitive areas. However, the variability in treatment schedules, therapy duration, and timing of surgery underscores the need for standardized protocols to optimize patient outcomes and facilitate comparison across studies.

The reported adverse events, while generally consistent with known immune-related toxicities, emphasize the importance of patient selection and monitoring, particularly for severe events [11]. The presence of fatal outcomes, albeit rare, highlights the need to balance therapeutic benefit with safety, especially in patients with pre-existing comorbidities.

### 4.1. Pathologic Response and Radiological Response

The weighted overall proportion for the pathologic response suggests the substantial potential of anti-PD1 in high-risk cSCCs, reinforcing the role of immunotherapy as a viable pre-surgical strategy for achieving significant tumor regression. However, the consistency across studies was described as having a large heterogeneity.

For the radiological response, the pooled estimate was slightly lower than the pathological response. A large heterogeneity in radiologic outcomes was observed across the included studies. This heterogeneity could arise from differences in imaging modalities or patients’ selection criteria, although studies with a higher number of patients were aligned in the application of RECIST 1.1 guidelines [26] to report objective imaging response [21,24,25].

Interestingly, discrepancies between radiological and pathological responses have been described as pathological complete responses exceeding the radiological complete response [21,24,25]. These findings underscore the challenges of relying only on imaging for treatment assessment and the importance of pathological evaluation, confirming previous observations also reported in neoadjuvant settings for melanoma management [27].

Additionally, the large heterogeneity observed in pathological and radiological responses indicates that factors influencing response rates, such as tumor biology, patient characteristics, and treatment timing, require further investigation.

### 4.2. Disease-Free Survival and Overall Survival

Neoadjuvant immunotherapy appears effective in achieving 1-year DFS and OS rates in advanced cSCC.

Data on 1-year DFS pooled proportion demonstrated excellent disease control after 1 year following neoadjuvant immunotherapy. Similarly, data on OS suggested the efficacy of neoadjuvant immunotherapy in prolonging survival.

The homogeneity in both 1-year DFS and OS indicated that these findings were consistently reported across different studies, although no significant outcome could be the consequence of the low number of included studies.

In conclusion, the high proportions observed for both 1-year DFS and OS underscore the promising role of neoadjuvant immunotherapy in advanced cSCC.

The overall findings in our systematic review emphasize the potential role of neoadjuvant immunotherapy in improving outcomes for patients with resectable cSCC, particularly in high-risk populations. The substantial pathologic response rates may translate into reduced recurrence and improved long-term survival.

### 4.3. Limitations

This analysis was limited by differences in the design of included studies. While larger studies provide high statistical evidence, smaller case reports may limit the generalizability of findings. Additionally, the reliance on published data introduces the potential for publication bias. The heterogeneity in sample size patient selection among the studies included in this meta-analysis introduces variability, which is important to consider when interpreting the pooled results. The predominance of male participants restricts the ability to draw conclusions on gender-specific responses to neoadjuvant immunotherapy. Adjunctively, survival data were only reported in a small number of studies and were found not to be significant despite the homogeneity across studies.

Further investigation is desirable to assess the durability of these responses and to explore combinations with other modalities, such as radiation therapy. In addition, comparative studies of neoadjuvant immunotherapy versus traditional approaches, such as surgery with adjuvant radiation therapy, would offer critical insights into its relative efficacy and safety.

Overall, these results support the growing role of ICIs in the neoadjuvant setting for cSCC but also point to critical areas for future research, including standardization of protocols, identification of predictive biomarkers, and long-term outcomes related to recurrence and survival.

## 5. Conclusions

This proportional meta-analysis demonstrated the promising efficacy of neoadjuvant immunotherapy in the management of resectable high-risk cSCC, with high pathological response rates and encouraging survival outcomes. The findings suggest that immune checkpoint inhibitors significantly reduced tumor burden, improving surgical outcomes and reducing extensive resections. The variability in treatment protocols highlights standardized approaches to optimize outcomes. While adverse events were generally consistent with known immune-related toxicities, further studies are needed to refine patient selection and minimize risks. Future research should focus on treatment standardization, long-term efficacy, and potential combinations with other therapeutic modalities to enhance disease control and patient quality of life. 

## Figures and Tables

**Table 1:** List of included articles and main findings.

Author (Year)	Study Design	Sample Size N	Male N (%)	Median Age	Disease Features	Drug Dosing Before Planned Surgery	Pathological Response Rate (Complete or Major)	Radiologic Response Rate (Complete or Partial)	1-Year Disease-Free Survival	Overall Survival	Adverse Effect(S)
Kim (2024) [25]	Retrospective study	27	18 (66)	72	Advanced cSCC	Cemiplimab, 350 mg Q3W from 2 to 4 doses Pembrolizumab 200 mg Q3W or 400 Q6W from 2 to 4 doses	47% (9 of 19)	50% (8 of 16)	90% (95% CI, 50.8–98.7%)	84% (95% CI, 51.2–95.9%)	No specific AEs reported; no patients discontinued therapy due to adverse effects
Gross (2022) [24], Gross (2023) [11]	Phase 2, multicenter, non-randomized study	79	67 (85)	73	Resectable stage II, III, or IV (M0) cSCC	Cemiplimab, 350 mg Q3W for up to four doses	50 (64%)	54 (68%)	92% (95% CI 82–97)	92% (95% CI 83–96)	≥Grade 3: 14 (18%)
Blaise (2023) [23]	Case report	1	1 (100)	66	SCC of the scalp and lymph node metastasis	Pembrolizumab, 200 mg Q3W for 4 doses	1 (100%)	-	-	-	None
Geidel (2023) [22]	Case report	1	1 (100)	55	SCC on the lower lip	Cemiplimab, 350 mg Q3W for 5 doses	1 (100%)		1 (100%)		No ≥ Grade 3
Ferrarotto (2023) [20]; Ferrarotto (2021) + erratum [21]	Single center, pilot phase II study	20	18 (90)	68.4	Resectable cSCC of head and neck stage III–IV	Cemiplimab 350 mg Q3W for 2 doses	15 (75%)	30% (6 of 20; 95% CI: 11.9–54.3)	89% (95% CI: 76.7–100)	95% (95% CI: 85.9–100)	No ≥ Grade 3
McLean (2022) [19]	Single-center retrospective study	6	4 (75)	65.5	Periorbital cSCC requiring orbital exenteration	Cemiplimab 350 mg Q3W Pembrolizumab 200 mg Q3W Up to 12 months	2 (100%)	4 (66%)	-	-	N/A
Goldfarb (2021) [18]	Single-center retrospective study	3	3 (100)	-	Locally advanced periorbital cSCC	Cemiplimab and Pemrbolizumab (no dosing reported)	2 (100%)	2 (66%)	2 (100%)	2 (100%)	N/A

Abbreviations: N, number; CI, confidence interval; Q3W, every 3 weeks; Q4W, every 4 weeks.

**Table 2:** Meta-analysis of the aggregated proportion of included studies.

	Sample Size	N. Event	Proportion (%)	95% CI
Pathologic response	137	89	72.2	57.7 to 84.6
Radiological response	135	79	54.8	38.6 to 70.5
One year disease-free survival	130	119	91.1	85.0 to 95.3
Overall survival	129	118	90.6	85.1 to 95.0

Abbreviations: N, number; CI, confidence interval.

## Data Availability

The data that support the findings of this study are available from the corresponding author upon reasonable request.

## Supplementary Materials

The following supporting information can be downloaded at: https://www.mdpi.com/article/10.3390/cancers17040637/s1.
